# Survey of extreme heat public health preparedness plans and response activities in the most populous jurisdictions in the United States

**DOI:** 10.1186/s12889-023-15757-x

**Published:** 2023-05-03

**Authors:** Nicole A. Errett, Cat Hartwell, Juliette M. Randazza, Amruta Nori-Sarma, Kate R. Weinberger, Keith R. Spangler, Yuantong Sun, Quinn H. Adams, Gregory A. Wellenius, Jeremy J. Hess

**Affiliations:** 1 Department of Environmental and Occupational Health Sciences, School of Public Health, University of Washington, 4225 Roosevelt Way NE, Suite 100, Seattle, WA 98105 USA; 2 Department of Environmental Health, Boston University School of Public Health, Boston, MA USA; 3 University of British Columbia, Vancouver, BC Canada; 4 Department of Global Health, University of Washington, Seattle, WA USA; 5 Department of Emergency Medicine, University of Washington, Seattle, WA USA

**Keywords:** Extreme heat, Public health, Preparedness, Policy, Climate change, Adaptation

## Abstract

**Background:**

Increasingly frequent and intense extreme heat events (EHEs) are indicative of climate change impacts, and urban areas’ social and built environments increase their risk for health consequences. Heat action plans (HAPs) are a strategy to bolster municipal EHE preparedness. The objective of this research is to characterize municipal interventions to EHEs and compare U.S. jurisdictions with and without formal heat action plans.

**Methods:**

An online survey was sent to 99 U.S. jurisdictions with populations > 200,000 between September 2021 and January 2022. Summary statistics were calculated to describe the proportion of total jurisdictions, as well as jurisdictions with and without HAPs and in different geographies that reported engagement in extreme heat preparedness and response activities.

**Results:**

Thirty-eight (38.4%) jurisdictions responded to the survey. Of those respondents, twenty-three (60.5%) reported the development of a HAP, of which 22 (95.7%) reported plans for opening cooling centers. All respondents reported conducting heat-related risk communications; however, communication approaches focused on passive, technology-dependent mechanisms. While 75.7% of jurisdictions reported having developed a definition for an EHE, less than two-thirds of responding jurisdictions reported any of the following activities: conducting heat-related surveillance (61.1%), implementing provisions for power outages (53.1%), increasing access to fans or air conditioners (48.4%), developing heat vulnerability maps (43.2%), or evaluating activities (34.2%). There were only two statistically significant (*p* ≥ .05) differences in the prevalence of heat-related activities between jurisdictions with and without a written HAP, possibly attributable to a relatively small sample size: surveillance and having a definition of extreme heat.

**Conclusions:**

Jurisdictions can strengthen their extreme heat preparedness by expanding their consideration of at-risk populations to include communities of color, conducting formal evaluations of their responses, and by bridging the gap between the populations determined to be most at-risk and the channels of communication designed to reach them.

**Supplementary Information:**

The online version contains supplementary material available at 10.1186/s12889-023-15757-x.

## Introduction

Extreme heat is the most fatal weather hazard in the United States, causing more deaths each year than all other weather-related disasters combined [1]. There is not one universally recognized definition of what constitutes an extreme heat event (EHE) or “heatwave,” but it is generally accepted to consist of a minimum of two to three days of unusually hot weather and often places additional stress on local infrastructure [2]. EHEs can lead to significant loss of life. For example, a heat event in Chicago in 1995 was responsible for 700 excess deaths [3], the 2003 European heatwave was associated with at least 70,000 deaths [4], and the 2021 Pacific Northwest and Western Canada heatwave has been associated with hundreds of deaths [5–9].

The frequency and intensity of EHEs are projected to continue increasing throughout the 21st century [10], exacerbating existing impacts of atmospheric warming. In fact, July 2021 was the warmest month ever recorded globally [11], and 2020 tied 2016 as the hottest years on record [12]. As such, communities across the globe are facing a looming public health crisis, requiring coordinated approaches to heat adaptation and extreme heat preparedness and response.

In addition to mortality, extreme heat is associated with emergency department visits and unplanned hospital admissions, increased cardiopulmonary and other diseases, negative pregnancy and birth outcomes, elevated issues of mental health, and higher health-care costs [13–18]. Some populations experience a higher risk of heat-related illness and death than others. The elderly are the most vulnerable group, while infants and children, low-income households, people with chronic medical conditions, and outdoor workers are also at an elevated risk of heat-related illness [2]. Disparities in heat-related illnesses by race and socioeconomic status have also been documented [19, 20].

Urban populations are increasingly vulnerable to the health impacts of heat as the interplay between climate change and the broader urbanization of the planet creates conditions that intensify warming [21]. More than half of the global population lives in cities today, with that number projected to reach 68% by 2050 [22]. North America is the most urbanized region in the world, as 82% of the population live in urban areas [22]. Rapid population growth in cities can lead to dense urban development, reducing vegetation and green space known to have a cooling effect [23]. The built environment, comprised of buildings, infrastructure and open spaces, influences urban temperatures [13]. The urban heat island (UHI) effect is the phenomenon in which urban temperatures are generally much higher than those in surrounding suburban and rural areas [23], increasing temperatures on average 5 to 15 °C relative to surrounding rural areas [24]. The UHI effect also produces temperature variations between neighborhoods in the same city, as differences in land cover characteristics such as vegetation, soil and water influence local temperatures [25, 26]. Simulations have suggested that concurrent power outages and EHEs will put 70–100% of the populations of major U.S. cities at risk for adverse health consequences [27]. With cities getting hotter because of the confluence of urbanization, UHI, and climate change, public health and emergency planners in jurisdictions with large populations across the U.S. must factor extreme heat and the potential for EHEs into their planning efforts [28].

**Fig. 1:**
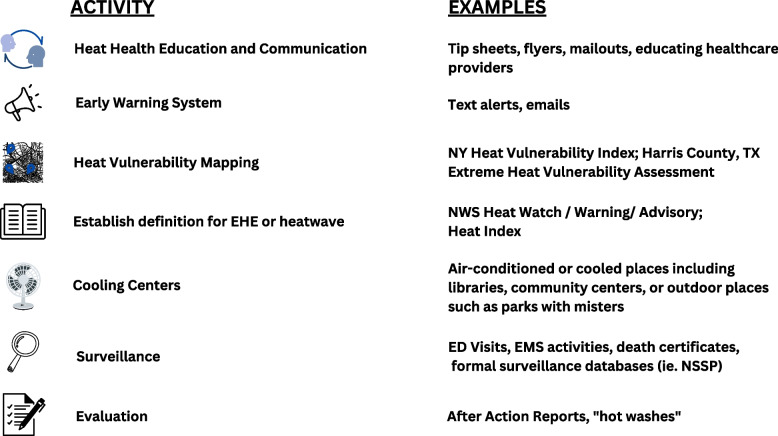
Common jurisdictional heat-related activities [2, 29, 33–38]

Public health and emergency management agencies may manage or participate in formal or informal heat early warning and response systems, which include a suite of communication and risk reduction strategies, in conjunction with weather forecasting, to limit population heat exposure and associated health impacts [29]. A 2011 survey of local health and emergency response departments found that the most common agency responses to extreme heat include risk communication, education and outreach to the public, collaboration with other organizations, and the opening of “cooling centers” – air-conditioned public spaces opened to the public during periods of extreme heat [30]. Only 7% of responding jurisdictions in that survey study reported having heat action plans (HAPs)—including both standalone plans and heat-specific components of all-hazards plans [30]. HAPs formally outline coordinated activities to prevent heat-related morbidity and mortality, providing guidance to government agencies for the provision of information and services to the public and targeted vulnerable populations during periods of extreme or dangerously high heat [31]. Figure 1 provides examples of common jurisdictional heat-related activities. An example of one such heat-related activity is heat vulnerability and/or social vulnerability mapping, by which jurisdictions create a map to visualize the spatial distribution of socio-economic conditions and environmental hazards so they can identify areas with higher risk of heat health outcomes [25]. Heat-related interventions, including HAPs are often triggered by forecasts from the National Weather Service (NWS) [31]. The NWS uses four tiers to categorize heat threats, including “excessive heat outlooks” which are issued 3 to 7 days ahead of a potential heat event; a “heat advisory” which is issued within a 12 hour period of the beginning of temperatures that are expected to be at least 100°F for 2 or more days and nighttime temperatures forecast to remain above 75°F; “excessive heat watches” which are issued when an excessive heat event is anticipated within 24 to 72 hours; and “excessive heat warning” which is the most severe of the tiers and is announced within 12 hours of the start of extremely dangerous heat when the heat index is forecast to be at least 105°F for two or more days and the temperatures at night will not fall below 75°F [31]. NWS categories serve as guidelines and are often adapted at the local level due to the variability of heat impacts across the country [32].

As EHE frequency and intensity is expected to increase in the coming years with disproportionate impacts in urban areas, the objective of this study is to assess the types and extent of activities that the most populated jurisdictions across the U.S. are engaging in to promote heat adaptation, to inform local extreme heat preparedness and response efforts and external support. The last survey study on this topic was conducted a decade ago [30]. Accordingly, this research provides a timely update of the status of heat preparedness and response implementation in the U.S.

## Methods

### Overview

A national survey was electronically distributed to U.S. jurisdictions in states with at least 200,000 residents from September 2021 to January 2022. Summary statistics were calculated to describe the types of extreme heat preparedness and response activities undertaken and to assess if the presence of a HAP was associated with the type of activities planned or performed. The University of Washington’s Human Subjects Division determined this research was not human subjects research, as defined by state and federal regulations and not subject to oversight by the University of Washington Institutional Review Board on August 18, 2020 (STUDY00010935).

### Survey design

A 97-question survey was developed based on the literature on extreme heat preparedness and response, and informed by a 2011 survey of heat preparedness and response at the county level, following a summer of record-setting heat across 30 states in the U.S [30]. Table 2 provides a description of common heat-related activities that were the focus of the survey questions (see Supplemental Material 1). The survey employed branching logic on questions pertaining to the presence of a written HAP, as well as those inquiring about specific heat-related activities such as issuing communications and having a working EHE definition (Supplemental Material 1). The original survey for our study was created in fall 2019, but this research was paused due to the unfolding coronavirus disease 2019 (COVID-19) pandemic in early 2020. The study resumed in late summer 2021, and the original survey questions were adjusted at that time to include questions about the impact of COVID-19 on extreme heat preparedness and response plans.

### Data collection

All jurisdictions with resident populations equal to or above 200,000 persons were identified for outreach based on 2019 Census data [39]. One hundred seventeen jurisdictions from across the 50 U.S. states, including both cities and counties, were initially identified. Notably, the U.S. local health and emergency management infrastructure is complex, and some jurisdictions engage in shared service arrangements where one agency provides services for multiple jurisdictions (e.g., the county health department also provides services for incorporated cities within or adjacent to its geographic footprint, such as in Miami-Dade County, which provides public health and emergency management services for the cities of Miami and Hialeah, both of which have populations over 200,000, and were identified for inclusion). Eighteen instances in which multiple cities shared services with the county level were identified, leaving a final sample of 99 unique jurisdictions for inclusion in our sample.

Two members of the research team (JR and CH) then conducted a manual search of the county/city websites to identify the emergency management agency and local health agency for each jurisdiction and, through their website or follow-up phone calls, identified names and contact information for the local emergency management director, local health officer, and local environmental health or public health preparedness director. In the event that individual-level contact information was unavailable, a generic agency email was collected.

Each jurisdiction was sent a single email to all identified points of contact, asking them to coordinate a single survey response on behalf of their jurisdiction. Two jurisdictions that had submitted responses to the original 2020 survey were recontacted separately and provided a PDF of their original response for reference with a request to update and resubmit a response. Four follow up emails were sent to all contacts in the database who had yet to respond between October until the survey closed in January 2022. The survey period was extended to maximize responses.

Respondents were offered a selection of books on climate change and public health emergency preparedness and response for their personal or organizational library to incentivize response.

Study data were collected and managed using REDCap electronic data capture tools hosted at the University of Washington’s Institute of Translational Health Sciences (ITHS) [40]. REDCap (Research Electronic Data Capture) is a secure, web-based application designed to support data capture for research studies, providing: 1) an intuitive interface for validated data entry; 2) audit trails for tracking data manipulation and export procedures; 3) automated export procedures for seamless data downloads to common statistical packages; and 4) procedures for importing data from external sources. REDCap at ITHS is supported by the National Center For Advancing Translational Sciences of the National Institutes of Health under Award Number UL1 TR002319.

### Analysis

Summary statistics were calculated in Microsoft Excel and R Studio Version 4.1.2 (R Core Team) to describe the proportion of total jurisdictions, as well as those with versus without heat action plans, that reported engagement in different extreme heat preparedness and response activities [41]. Basic summary statistics—including response rate and HAP status—were also computed for each of the ten U.S. Department of Health and Human Services (HHS) administrative regions [42] that reported engagement in different extreme heat preparedness and response activities. Fisher’s exact tests (one-sided) were used to evaluate the likelihood that jurisdictions with a reported HAP were more likely to engage in extreme heat activities compared to jurisdictions with no reported HAP.

While all respondents answered most questions, some respondents skipped certain questions leading to question-level missing data. To account for this in the analysis, jurisdictions that did not respond to the question about an existing written HAP (*n* = 4) were recoded as having no reported HAP. Jurisdictions that reported “Don’t know” for participating in heat activities were recoded as not participating in these activities. The decision to recode was made based on the assumption that jurisdictional engagement on a particular activity was unlikely to be occurring in a meaningful or coordinated way if the respondents, who were from the agencies responsible for EHE preparedness and response in their jurisdiction, were unaware or unsure of its implementation. Jurisdictions that did not respond to questions on participating in extreme heat activities were excluded from the analysis. Summary statistics and Fisher’s exact tests were calculated based on the total number of included responses at the question level. The total number of question-level responses (i.e., the denominator) used in analyses is indicated in Tables 1, 2 and 3.

**Table 1:** Survey response

**Response Rate** ***N* = 99**	**% (n)**
Survey response rate	38.4 (38)
**Jurisdiction Covered by Responding Agency** ***N* = 38**	**% (n)**
City	39.5 (15)
County	60.5 (23)
**Heat Action Plans** ***N* = 38**	**% (n)**
Has a written Heat Action Plan (HAP)	60.5 (23)
**Cooling Centers in HAP** ***N* = 23**	**% (n)**
Includes plans for cooling centers in written HAP	95.7 (22)

**Table 2:** Communications and activities in jurisdictions with and without written heat action plans (HAP)

	Overall *N* = 38 % (n)	Has HAP *N* = 23 % (n)	No HAP *N* = 15 % (n)	*P*-Value (Fisher’s)
**Communication Activities**				
Issues Communication Around Extrem Heat (*N* = 37)	100 (37)	100 (22)	100 (15)	-
Issues Comminications at Beginning of Summer (*N* = 38)	60.5 (23)	65.2 (15)	53.33 (8)	0.46
Issues Communications in Advance of Forecasted Heat Event (*N* = 38)	94.7 (36)	95.7 (22)	93.33 (14)	1
Issues Communications During Heat Event (*N* = 38)	84.2 (32)	87.0 (20)	80 (12)	0.36
Issues Communication in Different Languages (*N* = 38)	72.2 (26)	71.4 (15)	60 (11)	1
Issues Communications Directly to At-Risk Populations (*N* = 36)	41.7 (15)	47.6 (10)	33.33 (5)	0.50
**Other Heat-related Activities**				
Has Definition of Excessive Heat (*N* = 37)	75.7 (28)	90.9 (20)	53.3 (8)	0.01
Conducts Surveillance (*N* = 36)	61.1 (22)	77.2 (17)	35.7 (5)	0.02
Provides Extended Sheltering for People experiencing Homelessness During Heat Event (*N* = 37)	54.1 (20)	63.6 (14)	40.0 (6)	0.19
Has Provisions for Power Outages (*N* = 37)	53.1 9170	47.8 (11)	66.7 (6)	0.44
Increases Access to Fans/AC (*N* = 31)	48.4 (15)	43.5 (10)	62.5 (6)	0.43
Developed Vulnerability Heat Map (*N* = 37)	43.2 (16)	40.9 (9)	46.7 (7)	0.74
Conduct Evaluation (*N* = 38)	34.2 (13)	39.1 (9)	26.7 (4)	0.50
Performs Communications or Response Activities that Target At-Risk Populations (*N* = 27)	88.9 (24)	85.0 (17)	100 (7)	0.55

**Table 3:** Populations targeted for heat communication and platforms used to communicate

**At-risk populations targeted by plans include (*N* = 38)**	% (n)
Athletes	18.4 (7)
Elderly	55.3 (21)
People with pre-existing medical conditions	44.7 (17)
People with low incomes	86.8 (33)
Mobility challenged	36.8 (14)
People living in high-rise apartment buildings	10.5 (4)
Children	31.6 (12)
People working outdoors	42.1 (16)
People who live alone	34.2 (13)
Communities of color	31.6 (12)
People without A/C	39.5 (15)
People experiencing homelessness	50 (19)
Tourists	7.9 (3)
Non-English speakers	26.3 (10)
Undocumented people	10.5 (4)
**Platforms used to communicate heat warnings (*N* = 38)**	% (n)
Social media	97.4 (37)
News alerts	76.3 (29)
Internet	76.3 (29)
Press conferences	31.6 (12)
Joint events with other groups	34.2 (13)
Text alerts	31.6 (12)
Phone alert system	23.7 (9)
Flyers and posters	23.7 (9)
Email	42.1 (16)
Telephone hotlines	18.4 (7)
Door to door campaigns	13.2 (5)

## Results

### Survey response rate and heat action plans (HAPs)

38.4% (*n* = 38) of the 99 jurisdictions contacted responded to the survey. Two jurisdictions submitted duplicate responses, and were followed up with to determine the single response ultimately included. Of these 38 responses, 60.5% (*n* = 23) came from agencies at the county level and 39.5% (15) were from city agencies (Table 1). Nearly two-thirds of respondents, 60.5% (23) reported having a written HAP. The geographic distribution of survey responses according to the HHS administrative region is shown in Table 4.

**Table 4:** Geographic distribution of survey response and written heat action plans By HHS region

HHS Region	Total Jurisdictions Contacted	Response Rate of Contacted Jurisdictions % (n)	Respondents with Heat Action Plan % (n)
**Region 1**	*N* = 1	100 (1)	100 (1)
**Region 2**	*N* = 6	33.33 (2)	50 (1)
**Region 3**	*N* = 8	50 (4)	50 (2)
**Region 4**	*N* = 19	36.84 (7)	71.42 (5)
**Region 5**	*N* = 13	46.15 (6)	66.67 (4)
**Region 6**	*N* = 16	18.75 (3)	33.33 (1)
**Region 7**	*N* = 6	50 (3)	66.67 (2)
**Region 8**	*N* = 4	0 (0)	0 (0)
**Region 9**	*N* = 20	55 (11)	54.55 (6)
**Region 10**	*N* = 6	16.67 (1)	100 (1)

For jurisdictions reporting a written HAP, 4.3% (1) was created before 2001, 21.7% (5) were created between 2001 and 2010, 21.7% (5) were created between 2011 and 2020, and 4.3% (1) was created between 2021 and the present. The remaining 11 jurisdictions were unsure of the HAP origin date or did not answer. Of the 23 jurisdictions with HAPs, only 21.7% (5) reported that the written HAP was publicly available.

### Heat-related activities

All jurisdictions reported issuing emergency communications around extreme heat, including at the beginning of summer (60.5%, *n* = 23), in advance of forecasted EHE (94.7%, *n* = 36), and during heat events (84.2%, *n* = 32). Approximately three-quarters (72.2%, *n* = 26) reported communicating in different languages, and less than half identified that their jurisdictions issued communications directly to individuals from at-risk populations, for example directly contacting people in a registry (41.7%, *n* = 15) (Table 3).

Approximately three-quarters of respondents reported that their jurisdiction had developed a definition for what constituted an EHE (75.7%, *n* = 28). However, only two-thirds (61.1%, *n* = 22) reported conducting some type of heat-related surveillance, and only about half reported any provisions to: extend sheltering for people experiencing homelessness during high-heat days (54.1%, *n* = 20); address power outages, for example by providing relocation assistance or sheltering (53.1%, *n* = 17); or increase access to fans or air conditioners (48.4%, *n* = 15). Less than half of jurisdictions reported having developed a heat vulnerability map (43.2%, *n* = 16) or evaluating their extreme heat preparedness or response (34.2%, *n* = 13). EHE-specific health promotion activities were most commonly triggered automatically based on either NWS heat warnings or heat advisories (44.4%, *n* = 16), though some jurisdictions (38.9%, *n* = 14) reported that their activities were not triggered automatically by NWS heat warnings or heat advisories, and other reported activities being activated solely by NWS excessive heat warning (11.1%, *n* = 4) or solely by NWS heat advisories (5.6%, *n* = 2).

Jurisdictions with HAPs were more likely to have surveillance (*p* = 0.02) and a definition of extreme heat than jurisdictions without HAPs (*p* = 0.01); however no other statistically significant differences in the prevalence of EHE-related activities were identified between jurisdictions with and without a written HAP (Table 3).

Jurisdictions with HAPs were asked a subset of questions on their plans for cooling centers. Of the 23 jurisdictions with HAPs, 95.7% (22) reported establishing cooling centers as part of the activities covered in the plan (Table 1). Respondents reported that plans for cooling centers included “formal,” (47.8%, *n* = 11), defined as established and maintained by heat response programming, “informal,” (8.2%, *n* = 2), defined as established and maintained by community partners with no government oversight, or a mix of formal and informal (39.1%, *n* = 9) centers. About a quarter of respondents with plans for cooling centers (26.1%, *n* = 6) reported plans to provide transportation to established cooling centers during an EHE. Of the jurisdictions with plans for cooling centers in their HAPs, 43.5% (10) stated cooling centers were open for daytime hours, 13% (3) said they open for 24 hours, and 34.8% (8) described cooling center hours as “other.”

### At-risk populations and communication platforms

The vast majority (88.9%, *n* = 24) of survey respondents stated that their heat plans and activities include targeted strategies to communicate with or respond to the needs of at-risk or vulnerable populations. There was considerable variation in groups identified and targeted among responding jurisdictions (Table 3). People with low incomes were the most commonly targeted group (86.8%, *n* = 33). Older adults (55.3%, *n* = 21) and people experiencing homelessness (50%, *n* = 19) were the second and third most targeted populations. The top platforms used to communicate about heat are social media (97.4%, *n* = 37), news alerts (76.3%, *n* = 29), internet (76.3%, *n* = 29), and email (42.1%, *n* = 16) (Table 3).

## Discussion

As climate change drives the increased incidence of EHEs, public health and emergency management agencies are putting extreme heat preparedness and response plans and policies in place to protect the individuals they serve. However, we find that many populous US jurisdictions are taking piecemeal approaches to heat preparedness, with many lacking formal HAPs. While populations in urban jurisdictions are uniquely prone to health impacts of EHEs due to their built and social environments, over a third of the 38 U.S. jurisdictions with populations > 200,000 that responded to our survey reported not yet having developed a formal HAP. Climate impacts have shown that heat-related mortality and morbidity burdens in US cities require coordinated heat interventions to protect public health from the effects of extreme heat. Thus, our survey results suggest that many cities may need to bolster their efforts.

Our findings also affirm that jurisdictional approaches to extreme heat preparedness and response are highly divergent, with less than two-thirds of responding jurisdictions conducting any of the following activities: conducting heat-related surveillance (61.1%), implementing response provisions for power outages (53.1%), increasing access to fans or air conditioners (48.4%), developing heat vulnerability maps (43.2%) or evaluating extreme heat preparedness and response activities (34.2%). While risk communication was universally considered by responding jurisdictions, with the vast majority (88.9%) reporting targeting at-risk populations, methods of communication focused on passive, technology-dependent approaches that may exclude those most at-risk. For example, many jurisdictions identified people experiencing homelessness as being particularly vulnerable to extreme heat, but their communication outreach only included social media and dissemination through traditional news media, both of which may be disproportionately inaccessible to this population. These findings are suggestive of the need for additional guidance and resources to support jurisdictional extreme heat preparedness and response. Although the 2020 Centers for Disease Control and Prevention report “Heat Response Plans: Summary of Evidence and Strategies for Collaboration and Implementation” outlined potential components of a heat response plan and interventions [33], our findings suggest that additional guidance, dissemination efforts, training, and/or resources are likely necessary to support consistent planning approaches and widespread adoption of evidence-informed heat preparedness and response activities across U.S. jurisdictions.

To the best of our knowledge, no prior study has specifically assessed US urban jurisdictions’ extreme heat preparedness and response. However, a 2011 study examining county-level heat preparedness and response in 30 U.S. states provides some information to informally compare our findings and provide insights on national trends worthy of future exploration [30]. As these studies have significant differences in their design and study population, we are cautious in suggesting any direct comparisons. For instance, the 2011 survey focused specifically on the preparedness and response activities undertaken during the abnormally hot summer months of 2011, and included both urban and rural communities in their sample. Notwithstanding these differences, our findings indicate that there may be modest improvements in heat preparedness and response engagement over the past decade and/or differences across urban and rural jurisdictions, worthy of future investigation. Most notably, 60.5% of our study respondents reported having a HAP compared to 40% in 2011, 75.7% of our study respondents reported having a definition of excessive heat or heat wave compared to 30% in 2011, and 95.7% reported including cooling center plans in HAPs or opening cooling centers compared to 40% in 2011 [30]. Additionally, the jurisdictions in our study are incorporating social media, internet, emails, and news alerts more commonly into their communication plans compared to a decade ago [30].

Almost all of our respondents with HAPs (95.7%) reported plans to host formal or informal cooling centers. 26.1% of these respondents also reported providing transportation to cooling centers. Prior research based on qualitative interviews suggests that lack of or difficulty accessing transportation is a barrier to cooling center use [43]. This is particularly salient in the context of recent research by Adams et al. (2022), [44] which found that cooling centers may not be optimally located to serve populations at greatest risk of heat-related health effects [44]. We identified operational hours may pose another challenge for cooling center accessibility, as almost half of the jurisdictions with HAPs (43.5%, *n* = 10) said that cooling centers were only open during daytime hours and just 13.5% (*n*= 3) opened cooling centers for 24 hours. Previous research on cooling center accessibility in Maricopa County, Arizona, found that facility managers thought that operational hours were a potential constraint to preventing heat-related illness, as limiting hours to just daytime may not be optimal due to the dangers associated with exposure to nighttime heat [34]. The UHI effect exacerbates nighttime temperatures in cities and is strongest during the summertime, escalating overnight heat stress during warmer summer days [45]. Limiting cooling center hours to daytime hours may also limit access among heat-vulnerable populations such as outdoor workers who may not realistically be able to visit a cooling center during working hours [46]. While the cost of staffing cooling centers presents another operational obstacle [34], accessibility can be enhanced by extending the hours during an EHE or by pairing cooling centers with other strategies that address accessibility barriers, such as working with local businesses to open cooling facilities in areas of dense social vulnerability, increasing existing transportation, or adding options such as free shuttles to cooling centers [47, 48].

The field of public health emergency preparedness and response has been criticized for reliance on anecdotal information and an absence of evidence-informed practice [49]. Of concern, only about a third (34.9%) of jurisdictions report plans for evaluating their extreme heat preparedness and response activities, highlighting a missed opportunity to build this evidence base. Similarly, the 2011 survey of counties across U.S. states found that only 7% of respondents conducted an evaluation of their heat response [30]. Tools and resources to support evaluation that promote learning from and across jurisdictional experiences with extreme heat preparedness and response are urgently necessary to further understand the implementation and effectiveness of heat preparedness and response strategies, and should be developed and disseminated by federal partners (e.g., Centers for Disease Control and Prevention) and professional associations/membership organizations (e.g., the American Public Health Association or the National Association of County and City Health Officials). Further, grant funding requirements can incentivize jurisdictions to prioritize evaluative activities. While not specific to EHE preparedness and response, evaluation is emphasized in the CDC’s Building Resilience Against Climate Effects program and a required component of Climate-Ready States and Cities Initiative (CRSCI) grant recipient activities [50].

Vulnerability to heat is affected by geographic and socioeconomic factors, as well as more person-specific aspects such as gender, race and ethnicity, disability and health status [51]. Jurisdictions reported plans for heat communication and outreach that prioritized people with low-incomes, the elderly, and persons experiencing homelessness, which supports previous literature. For instance, age has repeatedly been cited as a risk factor for heat-related mortality, as has pre-existing conditions, living in poverty, working outdoors, and being socially isolated [25, 52–54]. Concurrently, urban heat exposures are an issue of environmental justice [55–57]. As a result of environmental racism and the legacy of redlining, communities of color and low-income populations disproportionately live in neighborhoods with less vegetation and tree cover, more intense UHI effect, and consequently greater heat exposures [55, 58]. As only 31% of respondents identified communities of color as an at-risk population, it is critical that jurisdictions apply an equity-focused lens as they develop and iterate upon their HAPs to protect populations that are disproportionately exposed to extreme heat and those that are more vulnerable to heat impacts.

The outlets by which jurisdictions communicate heat risk have evolved over the course of the 21st century. Research from the summers of 2004 and 2005 identified television, radio and newspaper to be the most common platform for heat risk communication at that time, with a high success rate of reaching the targeted audience [59]. In the following decade, digital technology has become more widely adopted, including by public agencies. Notably, we find discordance between the populations identified as being most at risk by respondents and the communication platforms most frequently used to reach the public, particularly the most vulnerable. Jurisdictions reported most commonly using passive, low cost channels of communications for extreme heat risk communication including social media, news alerts, the internet, and email. However, these platforms require access to and proficiency with technology that may act as a barrier for low-income people, older adults, and persons experiencing homelessness. At the same time, some of these same populations were also identified to be most at risk by the jurisdictions in our study. This discordance points to a need to consider alternative low-cost and active communications to better reach the most at-risk groups, and future research should investigate the efficacy of these programs as well as the effectiveness of other extreme heat preparedness and response activities. For example, New York City’s “Be A Buddy” program promotes social cohesion utilizing community volunteers to check on vulnerable residents during EHEs in heat vulnerable areas including the South Bronx, Central Brooklyn and Northern Manhattan [60]. “Be A Buddy” is a scalable and adaptable project that can be adopted by faith-based organizations, youth groups, and other community partners to expand the reach of messaging during extreme events in other communities.

### Limitations

While our study provides valuable preliminary insights about extreme heat preparedness and response activities among U.S. urban jurisdictions, its small sample size and unbalanced geographical distribution of respondents, with the West and South overrepresented and the Northeast and Central Northwest (HHS Districts 1 and 8) underrepresented (Table 4), may limit generalizability of study findings. Furthermore, it is possible that those who chose to respond to our survey may be the furthest along in extreme heat preparedness and response planning due to an elevated risk of EHE and/or having access to the necessary resources to plan. The ongoing COVID-19 pandemic may have affected survey completion and the response rate, as agencies continued to be overwhelmed. Additionally, the survey was administered during the fall and winter months, which is a period when heat may not be at the forefront of public health and emergency management priorities. Only 21.7% of jurisdictions with HAPs reported that their HAPs were publicly available, limiting researchers' ability to undertake more passive approaches to inventorying extreme heat preparedness and response activities, such as through analysis of plans posted online. Encouragement of formal sharing of HAPs could benefit the field by promoting inventories to use in evaluation, as well as sharing foundational examples, best practices, and innovative approaches. Finally, while our research explores EHE preparedness and response activities as reported by local health and/or emergency management agencies, U.S. jurisdictions are increasingly integrating specific programs, plans, and offices to accelerate climate action. Climate action plans (CAPs) are one such policy platform adopted by jurisdictions to incorporate climate change mitigation and adaptation strategies that are often longer term in scale, such as increasing vegetation and electrifying municipal vehicle fleets [61]. Future research should assess the types of EHE preparedness and response strategies contained in these plans, and if and how jurisdictions with CAPs differ in EHE preparedness compared to those without.

## Conclusion

As climate change intensifies heat, the most deadly weather-related hazard, so too does the need for calculated and coordinated preparedness and response. This need is particularly salient among urban areas, whose populations experience elevated risks to EHEs because of reduced tree cover and the UHI effect. Findings from this 2021 survey of 38 U.S. jurisdictions with populations of at least 200,000 reveal an urgent need to increase preparedness and response efforts and provide consistent guidance, tools, training, and resources to public health and emergency management planners. With only 60.1% of responding jurisdictions reporting adoption of formal HAPs, there is a clear opportunity for improvement. While all responding jurisdictions reported plans to communicate risk, responding jurisdictions diverged regarding their plans to conduct surveillance, respond to power outages, increase access to fans or air conditioners, develop heat vulnerability maps, or evaluate their activities. As jurisdictions develop and iterate upon their HAPs, it is critical that they apply an equity-focused lens in their plans to protect populations that are disproportionately exposed to extreme heat and those that are more vulnerable to heat impacts. Programs that engage in active, low-cost active communication and outreach are valuable and worth exploring, and future research should investigate the efficacy of these programs as well as the effectiveness of other extreme heat preparedness and response activities.

## Supplementary Information

**Additional file 1. **

## Data Availability

The survey instrument has been provided as supplemental materials. Datasets generated and/or analyzed during the current study are not publicly available due to concerns of privacy and confidentiality but are available from the corresponding author on reasonable request.

## Abbreviations

EHE: Extreme Heat Event
HAP: Heat Action Plan
HHS: Health and Human Services
ITHS: Institute of Translational Health Sciences
NWS: National Weather Service
REDCap: Research Electronic Data Capture
UHI: Urban Heat Island
