# Supplementary material for: Survey of extreme heat public health preparedness plans and response activities in the most populous jurisdictions in the United States
Source: BMC Public Health. 2023 May 3;23:811. doi: 10.1186/s12889-023-15757-x (PMC10154751; doi:10.1186/s12889-023-15757-x)
Supplement: Supplementary file 1 — Additional file 1. [file 12889_2023_15757_MOESM1_ESM.pdf]

Heat Action Survey

Please complete the survey below. Thank you!

What is the name of your jurisdiction?

____

What agency or agencies have contributed to the responses in this survey?

____

Within your jurisdiction, what is the name of the lead agency responsible for heat response?

____

Does this agency also oversee heat response for any other jurisdictions?

◯ Yes
◯ No

If yes, please list jurisdictions.

____

Jurisdiction's approach to excessive heat:

Is heat one of the hazards identified in your jurisdiction's all-hazards plan?

◯ Yes
◯ No
◯ Not sure

Has your jurisdiction conducted a needs assessment or related activity regarding heat in the last five years?

◯ Yes
◯ No
◯ Not sure

Is your jurisdiction currently addressing the health risks of heat with specific programming (for example, education and outreach, early warnings, risk reduction interventions, surveillance, or other specific activities)? Note: if your jurisdiction has a heat action plan, please check yes.

◯ Yes
◯ No

Does your jurisdiction have plans to develop heat programming?

◯ Yes
◯ No

What have been the barriers to developing heat programming in your jurisdiction? (Check all that apply)

☐ Budget
☐ Staff time
☐ Lack of heat hazard in our area
☐ Competing hazard priorities
☐ Competing programmatic priorities
☐ Other
☐ No barriers

Does your jurisdiction have a written heat action plan, policy, or procedure?

◯ Yes
◯ No

Please upload any relevant documents here, or email to heataction@uw.edu. We appreciate that some plans are confidential, and will keep the plan close-hold/not for further distribution upon request. In addition, we will not attribute any information contained in your plan to your jurisdiction in publicly facing papers, reports, or presentations if the plan is confidential.	
Is your plan publicly available?	◯ Yes<br>◯ No, the plan is confidential. Please do not share its contents beyond the study team or attribute any information contained in the plan to my jurisdiction in publicly facing papers, reports, or presentations.<br>◯ Other
If other, please describe.	__________________________________
What jurisdiction(s) are served by this heat action plan?	__________________________________
Does the health department have specific roles and responses outlined in the heat action plan?	◯ Yes<br>◯ No<br>◯ Not sure
What year did your jurisdiction first implement its plan/policy/procedure?	____________________________
When was the plan/policy/procedure last updated? (N/A if plan has not been updated)	____________________________
What were the major updates, if any?	____________________________
Is your plan/policy/procedure standalone (i.e. focused on heat) or part of an all-hazards plan?	◯ Standalone<br>◯ Part of an all-hazards plan<br>◯ Both<br>◯ Not sure
Does your heat action plan/policy/procedure capture all activities related to heat in your jurisdiction?	◯ Yes<br>◯ No<br>◯ Not sure
If no, please describe additional items that are not included in the policy:	__________________________________

What activities does your plan call for? Please check all that apply.

	Ongoing during the summer	Only during extreme heat events
Education regarding dangers associated with heat	☐	☐
Support for organizations providing services to reduce heat risks	☐	☐
Linkage of vulnerable populations with services to reduce heat risks	☐	☐
Direct services to reduce heat risks in vulnerable populations	☐	☐
Surveillance of heat-related morbidity and mortality	☐	☐
Evaluation of heat-related communication and programming	☐	☐
Interagency coordination regarding heat programming	☐	☐
Extended public park or public pool hours	☐	☐
Coordination with physicians/healthcare providers	☐	☐
Other	☐	☐

If other, please describe

____

In the jurisdiction(s) covered by the plan, are cooling centers established during extreme heat events?

◯ Yes
◯ No
◯ Not sure

Are the cooling centers formal (established and maintained by heat response programming) or informal (established and maintained by community partners with no government oversight)?

◯ Formal
◯ Informal
◯ Not sure
◯ Both

Does your jurisdiction provide transportation to cooling centers?

◯ Yes
◯ No
◯ Not sure

What are the approximate hours of the cooling centers during heat events?

◯ Daytime hours
◯ Overnight hours
◯ 24 hours
◯ Other

If other, please describe.

____

Do you collect information on the number of people who utilize cooling centers?	◯ Yes<br>◯ No<br>◯ Not sure
What factors do you consider when determining where to place cooling centers?	__________________________
Are your heat activities funded through your general funds or through a grant process that requires regular renewal?	◯ General funds<br>◯ Grant or other process requiring renewal<br>◯ Not sure
What is/are the prompt(s) for activating extreme heat event-based activities? Please check all that apply.	☐ NWS communication<br>☐ Pre-defined trigger from heat early warning system<br>☐ Directive or decision from the state health department<br>☐ Internal decision<br>☐ Change in syndromic or other surveillance data trends<br>☐ Other, please specify<br>☐ N/A, we have ongoing programming only
If other, please specify.	__________________________
Does your jurisdiction use the National Incident Management System (NIMS) or other incident management structure for any of its heat activity?	◯ Yes<br>◯ No<br>◯ Not sure

Jurisdiction's communication about extreme heat:

Does your jurisdiction issue communications about extreme heat?	◯ Yes<br>◯ No
When do you and/or your partners issue communications about heat? Please check all that apply.	☐ At the beginning of the summer<br>☐ In advance of a forecasted extreme heat event<br>☐ During an extreme heat event
Are any communications about heat made in multiple languages?	◯ Yes<br>◯ No<br>◯ Not sure
What platforms are used to communicate heat warnings? Please check all that apply.	☐ Social media<br>☐ News alerts<br>☐ Internet (for department, city, etc.)<br>☐ Press conferences led by health department<br>☐ Joint events with other groups<br>☐ Text alert system<br>☐ Phone alert system<br>☐ Flyers and posters<br>☐ Email messages<br>☐ Telephone hotlines<br>☐ Door-to-door campaigns<br>☐ Other
If other, please specify	__________________________

Are these communications targeted directly to individuals in specific at-risk populations? For example, directly contacting people in a registry.	◯ Yes<br>◯ No<br>◯ Not sure
Are any heat warnings made in multiple languages?	◯ Yes<br>◯ No<br>◯ Not sure

The definition of excessive heat:

Does your jurisdiction have a working definition for dangerous heat, a heatwave, or an extreme heat event?	◯ Yes<br>◯ No<br>◯ Not sure
Please include your definitions(s) here.	__________________________________
What is the source of the definition?	◯ NOAA/NWS<br>◯ State-wide definition<br>◯ City or county definition<br>◯ Other
If other, please specify.	____________________________
Does the definition incorporate any measures of humidity?	◯ Yes<br>◯ No<br>◯ Not sure
Does your definition include one more threshold(s) for temperature or related variables?	◯ One threshold<br>◯ Multiple thresholds<br>◯ Not sure
Please describe the threshold(s) your jurisdiction uses.	__________________________________
Please describe the different levels of activities your jurisdiction engages in at the different thresholds.	__________________________________

Coordination with meteorological agencies:

Does your jurisdiction receive meteorological data from the National Weather Service?	◯ Yes<br>◯ No<br>◯ Not sure
Does your jurisdiction receive meteorological data from other sources?	◯ Yes<br>◯ No<br>◯ Not sure
Do you receive raw data, interpretations, or both?	◯ Raw data<br>◯ Interpretations<br>◯ Both

How do heat event-specific health promotion activities within your heat action plan get activated?	◯ Automatically based on NWS excessive heat warning<br>◯ Automatically based on NWS heat advisories<br>◯ Automatically based on either NWS excessive heat warnings or heat advisories<br>◯ Neither automatically based on NWS excessive heat warnings or heat advisories
If neither, please explain how activities are activated:	__________________________________
Please describe any circumstances in which this might occur:	__________________________________

Outdoor event cancellation

Does your jurisdiction have an extreme heat outdoor event modification/delay/cancellation policy?	◯ Yes<br>◯ No<br>◯ Not sure

Cascading failures/Utilities

Does your jurisdiction's heat plan, policy, or procedure have provisions for power outages during a heat event (for example, relocation assistance or sheltering)?	◯ Yes<br>◯ No<br>◯ Not sure<br>◯ We do not have an excessive heat plan
Does your jurisdiction institute a moratorium on disconnecting utilities during heat events?	◯ Yes<br>◯ No<br>◯ Not sure
Does your jurisdiction institute a moratorium on disconnecting utilities during the summer?	◯ Yes<br>◯ No<br>◯ Not sure
Does your jurisdiction's heat plan include provisions to increase access to personal fans/air conditioners?	◯ Yes<br>◯ No<br>◯ Not sure<br>◯ We do not have an excessive heat plan
Does your jurisdiction's heat plan include provisions for helping people pay their utility bills or other forms of heat-related energy assistance?	◯ Yes<br>◯ No<br>◯ Not sure<br>◯ We do not have an excessive heat plan
How is this financial support provided? Check all that apply.	☐ Fuel assistance program funds<br>☐ Private donations<br>☐ Government funds<br>☐ Other
If other, please describe.	__________________________________

Vulnerable Populations and Equity

Does your jurisdiction's heat plan have specific communications or response activities for at-risk or vulnerable populations?

- ◯ Yes
- ◯ No
- ◯ Not sure
- ◯ We do not have an excessive heat plan

What populations are targeted? Check all that apply

- ☐ Athletes
- ☐ Elderly
- ☐ People with pre-existing medical conditions
- ☐ People with low incomes
- ☐ Mobility challenged
- ☐ People living in high-rise apartment buildings
- ☐ Children
- ☐ People working outdoors
- ☐ People who live alone
- ☐ Communities of color
- ☐ People without air conditioning
- ☐ People who are experiencing homelessness
- ☐ Tourists
- ☐ Non-English speakers
- ☐ Undocumented people
- ☐ Other

If other, please describe.

____

Has a heat vulnerability map been developed for your jurisdiction?

- ◯ Yes
- ◯ No
- ◯ Not sure

How is that map used in your heat plan, policy, or procedure, and response efforts?

____

Does your jurisdictions provide extended sheltering for people who are experiencing homelessness during heat emergencies?

- ◯ Yes
- ◯ No
- ◯ Not sure

Epidemiologic Surveillance

Does your jurisdiction have health surveillance activities related to heat?

- ◯ Yes
- ◯ No
- ◯ Not sure

What data sources are used to monitor the health impacts of excessive heat? Check all that apply.

- ☐ Hospital admissions
- ☐ Emergency department visits
- ☐ Social media
- ☐ Emergency Medical Service use
- ☐ Death certificates
- ☐ Not sure
- ☐ Other

If other, please describe.

____

What health outcomes are tracked? Check all that apply.	☐ All-cause mortality<br>☐ Heat-related illness (ex heat exhaustion, heat stroke)<br>☐ Cardiac admissions<br>☐ Respiratory admissions<br>☐ Renal admissions<br>☐ Cerebrovascular admissions<br>☐ Injuries<br>☐ Drownings<br>☐ Not sure<br>☐ Other
If other, please describe.	__________________________________

Longer-Term Adaptation

Some jurisdictions are implementing long-term actions to adapt to excessive heat. Please check any of the actions below that your jurisdiction is undertaking.	☐ Green roofs<br>☐ Cool pavements<br>☐ Tree planing/increased green space<br>☐ A law/regulation governing maximum permissible temperature for residential buildings<br>☐ A law/regulation banning landlords from charging extra for tenant use of air conditioning<br>☐ A law/regulation to allow tenants/homeowners to install air conditioning<br>☐ Not sure<br>☐ Other
If other, please describe.	____

Program Evaluation

Does your jurisdiction formally evaluate its approach to excessive heat?	◯ Yes<br>◯ No<br>◯ Not sure
How frequently is an evaluation effort undertaken for your heat plan, policy, or procedure?	◯ Afer every heat event<br>◯ Every year<br>◯ Every 2-5 years<br>◯ Unsure<br>◯ Other
If other, please describe.	__________________________________

COVID-19 and Extreme Heat

Did you modify your approach to managing extreme heat because of COVID-19?

- ◯ Yes
- ◯ No

Did you approach to implementation change due to COVID-19 for any of the following activities? (Check all that apply)

- ☐ Education regarding dangers associated with heat
- ☐ Support for organizations providing services to reduce heat risks
- ☐ Linkage of vulnerable populations with services to reduce heat risks
- ☐ Direct services to reduce heat risks in vulnerable populations
- ☐ Surveillance of heat-related morbidity and mortality
- ☐ Evaluation of heat-related communication and programming
- ☐ Interagency coordination regarding heat programming
- ☐ Extended public park or public pool hours
- ☐ Coordination with physicians/healthcare providers
- ☐ Other (please describe)

If you selected any of the activities or "other", please describe how implementation changed.

____

If your heat plan was modified due to COVID-19, please describe how.

____

Conclusion

What priority is heat in your jurisdiction?

- ◯ Low priority
- ◯ Medium priority
- ◯ High priority

What resources or sources of data do you need to better understand or evaluate the impact of heat in your community?

____

As a token of our appreciation, we are offering participants who complete the survey copy of a climate change and/or public health-related book for their personal or organizational library (while supplies last). Book options include The Great Influenza by John M. Barry, The Unthinkable by Amanda Ripley, Five Days at Memorial by Sheri Fink, Heat Wave: A Social Autopsy of Disaster in Chicago by Eric Klinenberg, A Fire Story by Brian Fies, Fire in Paradise: An American Tragedy by Alastair Gee and Dani Anguiano, and The Ghost Map by Steven Johnson. If you are interested in receiving a book, please indicate your preferred book and include your complete shipping address. Please note that we are only accepting one survey submission per health department, and thus are only able to provide one book per participating health department.

____

Would you be willing to participate in a follow-up interview to provide information about the implementation of your LHJ's heat response program or activities? If you would be willing to participate in a follow-up interview, please provide your email address. __________________________________
